# Genotypic identification of *Panicum* spp. in New South Wales, Australia using DNA barcoding

**DOI:** 10.1038/s41598-021-95610-6

**Published:** 2021-08-06

**Authors:** Yuchi Chen, Xiaocheng Zhu, Panayiotis Loukopoulos, Leslie A. Weston, David E. Albrecht, Jane C. Quinn

**Affiliations:** 1 School of Agricultural, Environmental and Veterinary Sciences, Charles Sturt University, Wagga Wagga, NSW Australia; 2 Graham Centre for Agricultural Innovation, Charles Sturt University and NSW Department of Primary Industries, Wagga Wagga, NSW Australia; 3 Melbourne Veterinary School, The University of Melbourne, Werribee, VIC Australia; 4 Wagga Wagga Agricultural Institute, NSW Department of Primary Industries, Wagga Wagga, NSW Australia; 5 Australian National Herbarium, Centre for Australian National Biodiversity Research (a Joint Venture Between Parks Australia and CSIRO), Canberra, Australian Capital Territory, Australia

**Keywords:** Molecular biology, Plant sciences

## Abstract

Australia has over 30 *Panicum* spp. (panic grass) including several non-native species that cause crop and pasture loss and hepatogenous photosensitisation in livestock. It is critical to correctly identify them at the species level to facilitate the development of appropriate management strategies for efficacious control of *Panicum* grasses in crops, fallows and pastures. Currently, identification of *Panicum* spp. relies on morphological examination of the reproductive structures, but this approach is only useful for flowering specimens and requires significant taxonomic expertise. To overcome this limitation, we used multi-locus DNA barcoding for the identification of ten selected *Panicum* spp. found in Australia. With the exception of *P. buncei*, other native Australian Panicum were genetically separated at the species level and distinguished from non-native species. One nuclear (*ITS*) and two chloroplast regions (*matK* and *trnL* intron*-trnF*) were identified with varying facility for DNA barcode separation of the *Panicum* species. Concatenation of sequences from *ITS, matK* and *trnL* intron*-trnF* regions provided clear separation of eight regionally collected species, with a maximum intraspecific distance of 0.22% and minimum interspecific distance of 0.33%. Two of three non-native *Panicum* species exhibited a smaller genome size compared to native species evaluated, and we speculate that this may be associated with biological advantages impacting invasion of non-native *Panicum* species in novel locations. We conclude that multi-locus DNA barcoding, in combination with traditional taxonomic identification, provides an accurate and cost-effective adjunctive tool for further distinguishing *Panicum* spp. at the species level.

## Introduction

*Panicum* represents one of the largest genera of the Poaceae, and species are widely distributed globally from the subtropics to temperate regions^1^. Up to 500 species are recognised worldwide, depending on the taxonomic system adopted^1,2^. *Panicum* species inhabit temperate, semi-arid, arid and tropical environments in Australia, encompassing a range of shady or open habitats including forests, woodlands, grasslands, wetlands and variously disturbed sites including cultivated fields^1,2^. The greatest numbers of distribution records of *Panicum* species in Australia are from eastern and northern Australia^3^. To date, 24 indigenous and nine non-native species of *Panicum* were identified in Australia (Council of Heads of Australasian Herbaria 2005- onwards, Australian Plant Census).

Currently, *Panicum* grasses are identified as economically important weeds of summer fallow pastures in Australia^4^. Additionally, *Panicum* grasses are also widely recognised as a common causative agent of crystal-associated cholangiohepatopathy in herbivores worldwide^5,6^, and are the most commonly identified species associated with hepatogenous photosensitisation in Australian livestock^7^. Hepatotoxicity related to the ingestion of *Panicum* grass species is clearly associated with the effects of saponins or sapogenins present within this genus^8^. Characterisation of steroidal saponins has not been undertaken for all *Panicum* species found in Australia or elsewhere^9^, however, previous reports have suggested that saponins or sapogenin profiles differ between species^10^. It was postulated that diverse chemical profiles may be associated with differential toxicity in livestock related to the ingestion of different *Panicum* species^10^. Therefore, accurate and reliable identification of the *Panicum* spp. is critical for effective management, pasture monitoring, livestock disease investigation, and chemical profiling.

**Figure 1:**
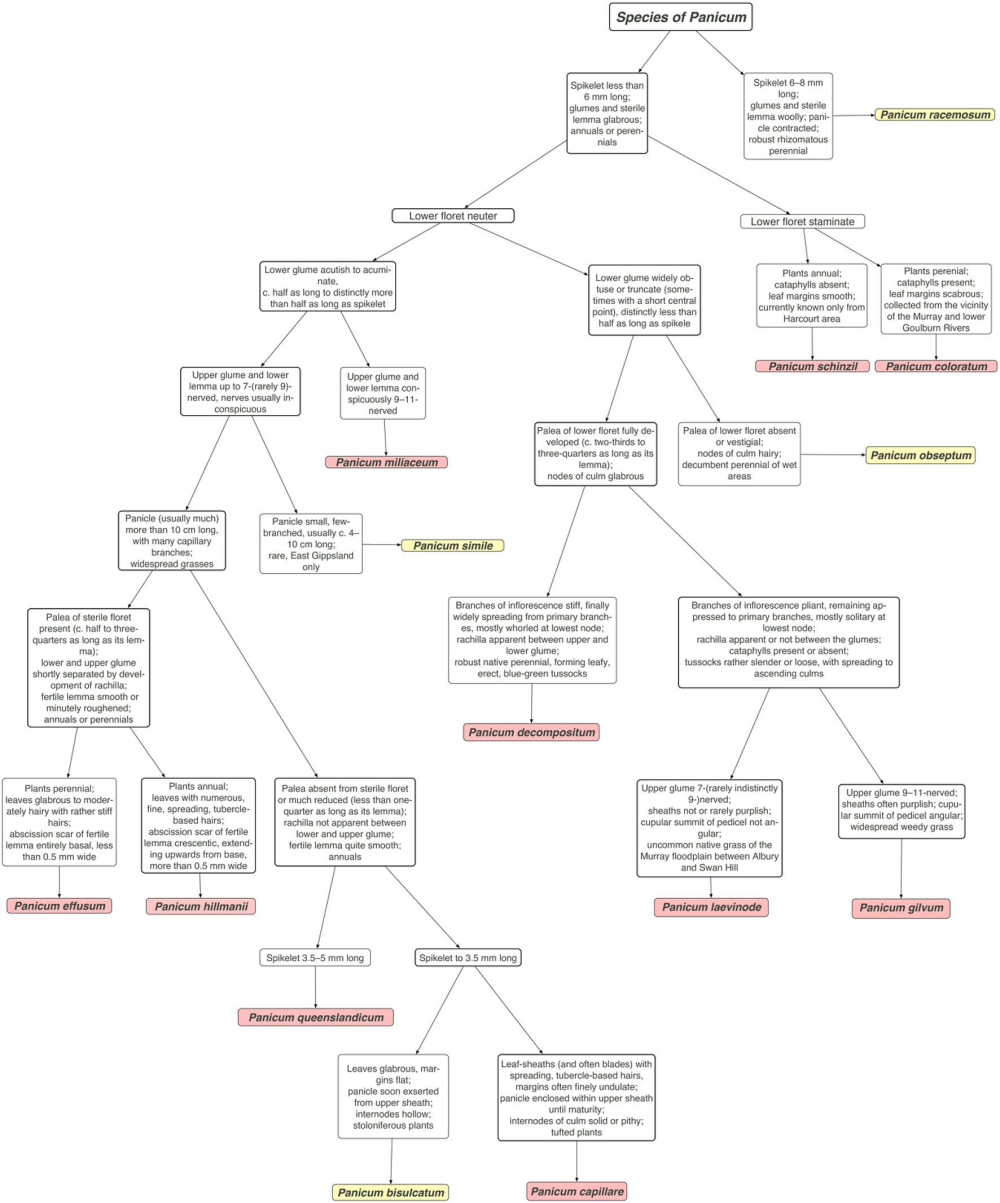
Taxonomic key for differentiation of selected *Panicum* species. Species included in this study are highlighted in pink, and other species are highlighted in yellow. ^11^ Modified from Walsh and Entwisle.

Traditionally, morphological features were used to differentiate *Panicum* spp. (Fig. 1)^11^. However, species identification based on morphology is not a trivial task as morphological differences between species can be subtle, even when considering native and non-native species^12,13^. A microscope is frequently needed to observe critical features such as the shape of the abscission scar at the base of the fertile lemma. Morphological keys to species are also heavily biased towards reproductive characters thereby rendering identification of sterile specimens difficult, if not impossible, even for a grass specialist. Although precise identification is possible using morphological keys, especially if reproductive material is available^14^, successful usage of these keys requires a clear understanding of morphological structures and a proficiency in using keys. For example, the taxonomic key to differentiate *Panicum effusum* R.Br. (native to Australia) and *P. hillmanii* Chase (introduced to Australia from North America) is based on the shape of the abscission scar of the fertile lemma. The abscission scar of the fertile lemma of *P. effusum* is entirely basally located and less than 0.5 mm wide while *P. hillmanii* has a crescentic abscission scar of the fertile lemma, extending upwards from the base, and is more than 0.5 mm wide^15^. The level of expertise required to detect minute morphological differences presents a major challenge for the inexperienced and examination by a grass taxonomist may ultimately be required for consistency in identification.

Molecular technologies are increasingly used to develop reliable methods for plant and animal species identification^16^. A PCR-based genotyping method, DNA barcoding, has been extensively applied for this purpose^17^. DNA barcoding is a method that uses short but informative standardised DNA regions ("barcodes") to identify or differentiate between species^18–20^. It was first proposed in 2003^17^, and was utilised as an important complementary method to traditional morphological identification^21^, for vegetation and floristic surveys^22^, ecological forensics^23^, regulatory enforcement^24,25^, community phylogenies, comparative biology and phylogenetic diversity^26^. Selection of the "barcode" is critical to establish a successful DNA barcoding platform to identify *Panicum* species. An ideal barcode should be a short DNA sequence that can be routinely amplified using a standard PCR method. The amplified product should also be easily sequenced with universal primers that are anchored in highly conserved DNA regions, and the sequences should be easily aligned without extensive manual editing^22^. Most importantly, these regions should be able to differentiate between the target species^18^. However, unlike animals where the sequence for cytochrome oxidase 1 (*CO1*) in mitochondrial DNA was proposed as the universal barcode for species identification^17^, the identification of an universal barcode for many plant species, and *Panicum* in particular, remains challenging due to inter-species mutation and technical reliability^27^. Unfortunately, *CO1* is not suitable for use in plants as the nucleotide substitution rate within mitochondria in plant cells is relatively low^28^. Additionally, there has been difficulty in locating highly heterogeneous regions in plant DNA due to a lack of sequence polymorphism, slow mutation rates^29^, frequent introgression or species hybridisation between related species^30^, and incomplete lineage sorting^22^.

To overcome these issues, a multi-locus approach for plants was demonstrated to improve identification capability and reliability^18^. Multiple barcoding studies have further suggested that a combination of *rbcL* and *matK* sequences are suitable for DNA barcode GAP analysis in *Panicum* spp^31–35^. Moreover, the use of the chloroplast gene *ndhF*, alone or in combination with *rbcL* and *matK*, has been proposed^32,36^. Additionally, the use of the *ndhF* region may also increase the resolution level when used to discern between grass species^37,38^. Unfortunately, the use of *trnH-psbA* for differentiating *Panicum* species has not proven useful, as the existence of inversions or mononucleotide repeats at this locus can result in incorrect alignments or additional difficulties in sequencing^39^. To date, the nuclear ribosomal Internal Transcribed Spacer (*ITS*) locus has not been used as a species discriminating barcode in *Panicum* spp., but it has been proposed that *ITS* is a suitable marker for genetically similar species and could be used as a core or complementary barcode^40^. Currently, the optimal suite of barcoding loci has not yet been fully established for identification of various *Panicum* species in Australia. Therefore, this study has focused on the use of the nuclear locus *ITS* as the core barcode for genotypic identification of ten native and non-native *Panicum* species found in southern New South Wales, together with two plastid loci, *matK* and *trnL* intron*-trnF* as complementary loci^41^.

The establishment of a robust and objective method for identification using both genetic markers and morphological traits is required to address and overcome the challenge of differentiation of *Panicum* species in both field monitoring and laboratory studies and would enable unambiguous identification of field samples collected at any stage of the plant’s growth cycle. To achieve this outcome, we developed and validated a DNA barcoding method for identification and differentiation in ten species of *Panicum* that are frequently found in south-eastern Australia. This study also tested the hypothesis that *Panicum* species with a smaller genome size have a greater potential to become invasive in a novel environment^42^, by determination of the genome size of several indigenous and non-native *Panicum* species in southern New South Wales.

## Results

### Sampling

**Figure 2:**
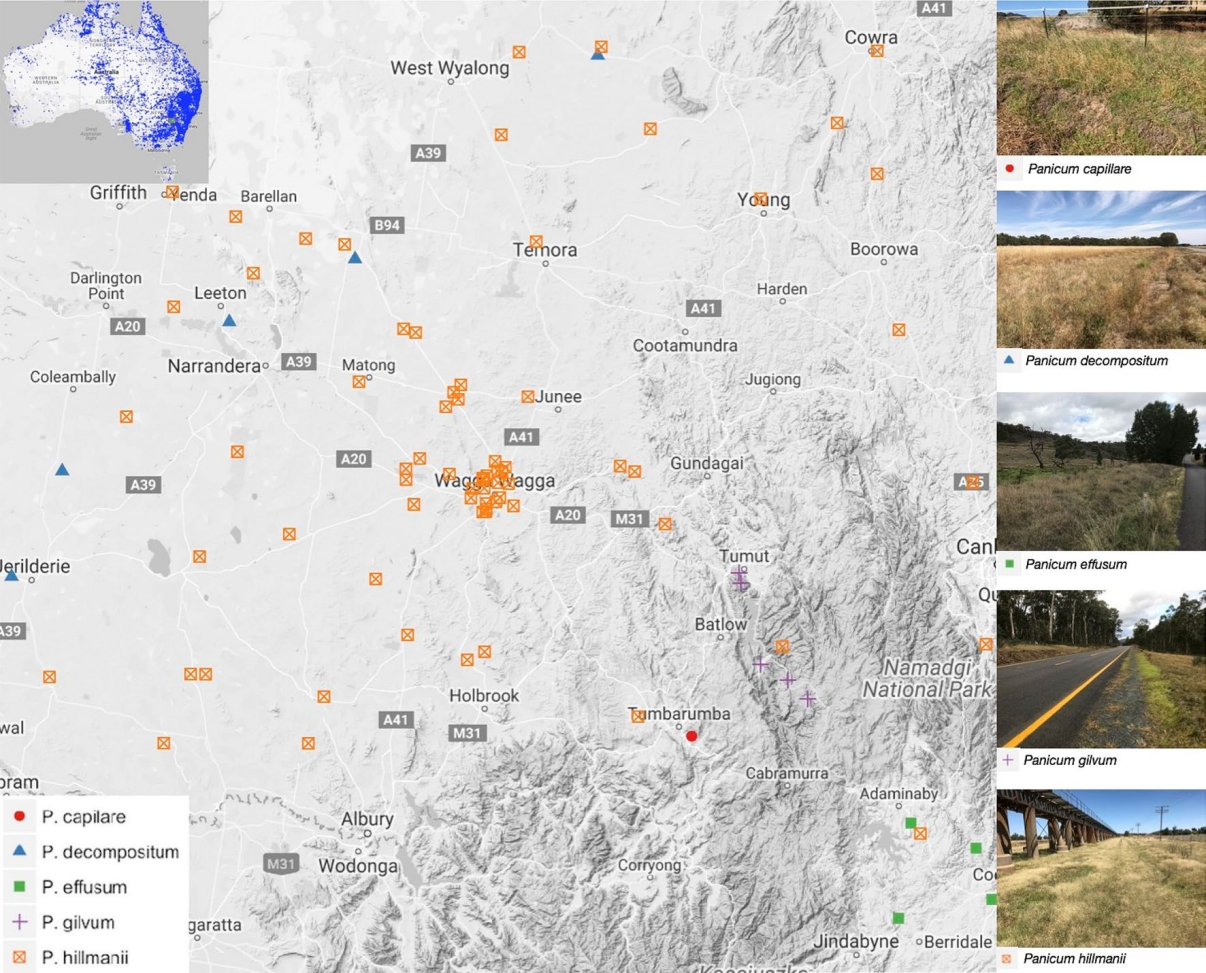
Location of field collected *Panicum* spp. within a 200 km radius of Wagga Wagga, New South Wales, Australia. Right: representative photos of collected plants in their habitats. Inset map: Distribution of recorded *Panicum* species in Australia (Australia Virtual Herbarium Database, 2019).

*Panicum* plants (106 individuals) were sampled from geographically dispersed locations within a 200 km radius of Wagga Wagga, New South Wales, Australia (Fig. 2). Morphological examination of these specimens at the Australian National Herbarium (CANB) revealed that five *Panicum* species were captured by field sampling. To bolster the number of species included in the DNA barcode GAP analysis, sampling of herbarium specimens held by CANB was undertaken. A total of 40 samples (17 field samples and 23 herbarium samples), representing ten indigenous and non-native *Panicum* species, were included in the analysis (Table 1).

**Table 1:** Origin and number of *Panicum* species subjected to DNA barcoding analysis and derived from field site or herbarium collections.

Species	Native or non-native	Country of origin^45^	Number of field samples	Number of herbarium samples
*P. buncei* F. Muell	Native	Australia	0	2
*P. coloratum* L	Non-native	Africa	0	2
*P. capillare* L	Non-native	North America	1	2
*P. decompositum* R.Br	Native	Australia	3	5
*P. effusum* R.Br	Native	Australia	4	1
*P. gilvum* L	Non-native	Africa	5	0
*P. hillmanii* Chase	Non-native	North America	4	2
*P. laevinode* Lindl	Native	Australia	0	3
*P. miliaceum* L	Non-native	Asia	0	3
*P. queenslandicum* Domin	Native	Australia	0	3

Each species was identified as native or non-native, with continent of origin indicated.

### DNA barcode gap analyses

PCR amplification and sequencing were undertaken for all samples for the three selected regions: *ITS*, *matK* and *trnL* intron-*trnF*. Sequenced loci of these three regions were submitted to GenBank and their accession numbers were listed after the specimen’s name in the phylogenetic tree. Alignments of each region were truncated to 641, 730, and 750 bp for *ITS, matK* and *trnL* intron*-trnF*, respectively. Concatenated loci, one nuclear locus with either one or two plastid loci, were calculated for barcoding gaps. Further intraspecific and interspecific distance analyses were performed on eight *Panicum* species (Table 2). *P. buncei* (native) and *P. coloratum* (non-native) were not included in these analyses as they were not genetically separated by any of the three regions).

**Table 2:** Intraspecific and interspecific K2P distances for the three gene loci *ITS*, *matK*, *trnL* intron*-trnF* in eight *Panicum* species.

Species max ID %	Loci				*ITS* + *matK* + *trnL* intron*-trnF*
	*ITS*	*matK*	*trnL* intron*-trnF*	*ITS* + *matK*	
Min ID %	0.71	0.07	0	0.61	0.33
*Panicum capillare*^a^	0.33	0.09	0	0.33	0.22
*P. decompositum*	0.03	0.06	0	0.05	0.03
*P. effusum*	0.34	0	0	0.22	0.15
*P. gilvum*^a^	0	0	0	0	0
*P. hillmanii*^a^	0	0	0	0	0
*P. laevinode*	0.07	0.09	0	0.09	0.06
*P. miliaceum*^a^	0	0.09	0	0.05	0.03
*P. queenslandicum*	0.14	0.14	0.27	0.09	0.16

*Panicum buncei* and *P. coloratum* were not included in this table because of overlap in respective DNA barcodes. Minimum interspecific distance, MinID; Maximum intraspecific distance, MaxID.

Non-native species are denoted with ^a^.

**Figure 3:**
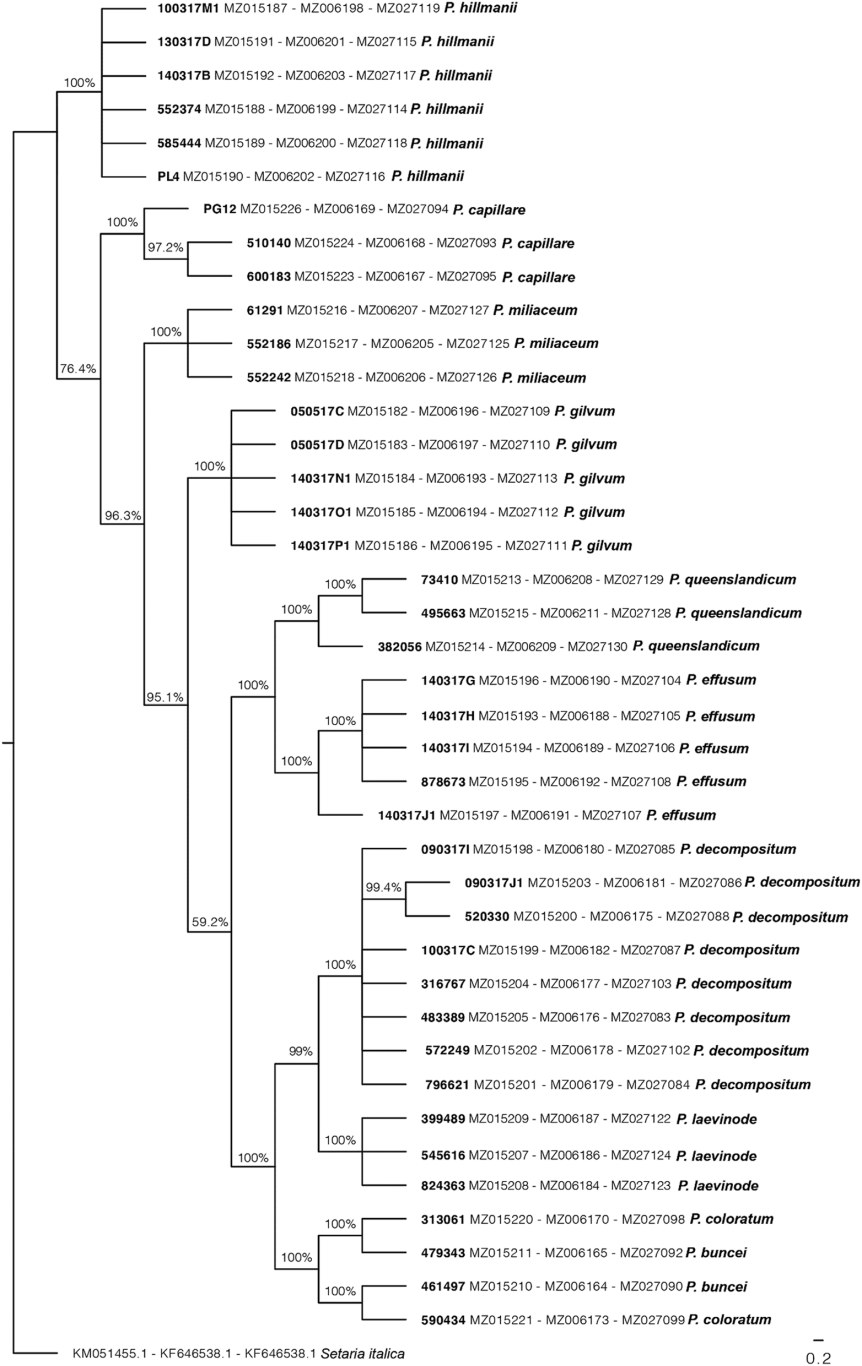
Bayesian phylogenetic relationships among ten *Panicum* species inferred from the concatenation of three conserved genetic sequences. Species ID on the terminal node was shown as voucher number GenBank accession number (*ITS-MatK- trnL* intron *trnF*) and species name. Clade posterior probability is indicated at nodes. Accession identifiers are shown in grey.

Phylogenetic tree inferred using Bayesian inference clustered most species into highly supported clades (Fig. 3). All native species (*P. effusum*, *P. queenslandicum*, *P. decompositum*, *P. laevinode*, *P. buncei*) were clustered into a large group, although the posterior probability was low (59%). The majority of the non-native species (*P. hillmanii*, *P. capillare*, *P. miliaceum* and *P. gilvum*) was clustered into clades separated at the species level. Most species (both native and non-native) were classified into monophyletic groups. Exceptions included the non-native species *P. coloratum*, which clustered with the native *P. buncei*.

### Determination of genome size in non-native and Australian native *Panicum* species

**Figure 4:**
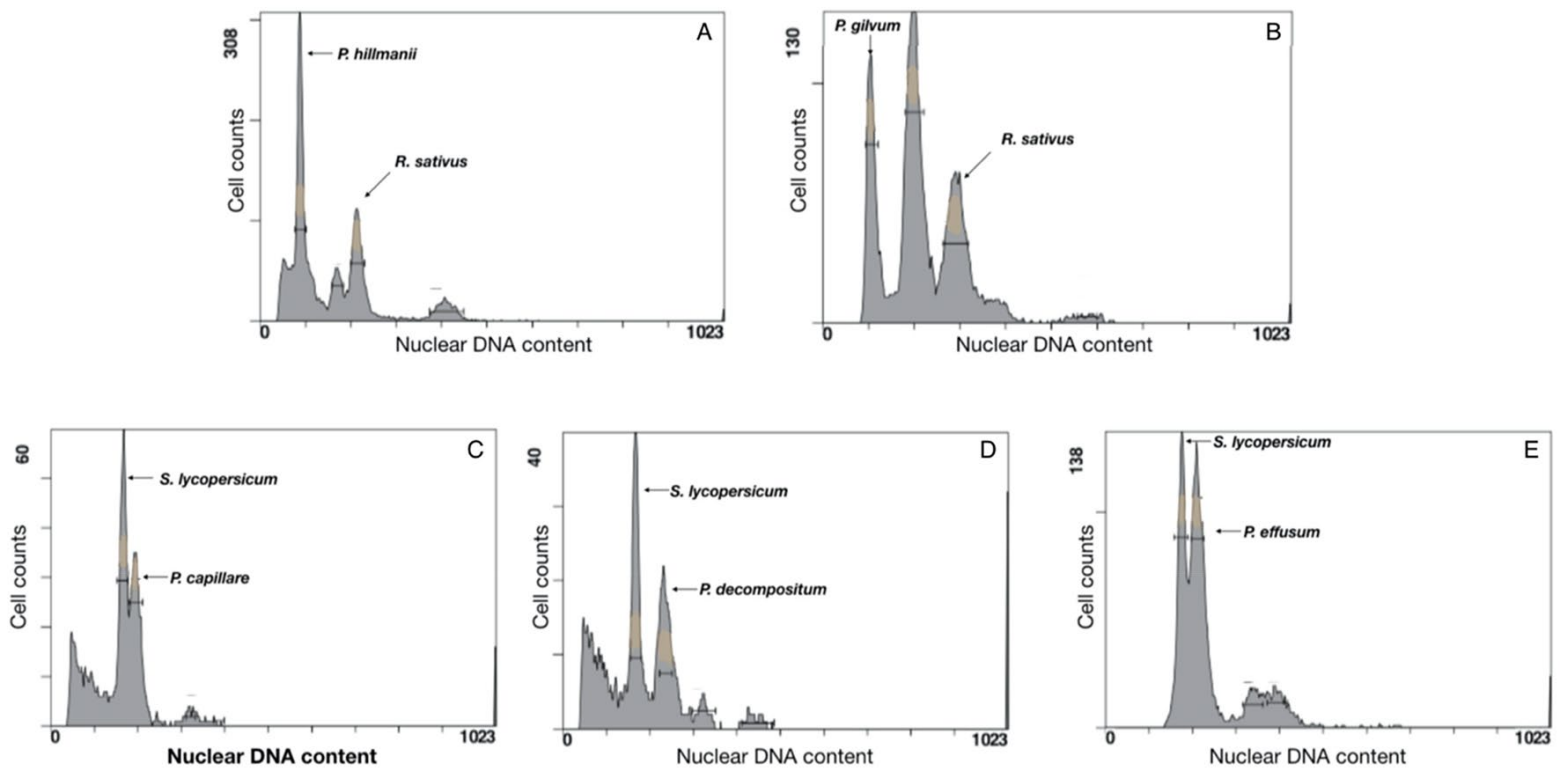
Flow cytometry histograms of *Panicum hillmanii* (**A**) and *P. gilvum* (**B**) using radish (*Raphanus sativus*, 1C = 0.55 pg), together with *P. capillare* (**C**), *P. decompositum* (**D**) and *P. effusum* (**E**) using tomato (*Solanum lycopersicum,* 1C = 1.06 pg), as an internal reference.

To investigate the genome size of each species, and the associated hypothesis that genome size is linked to success in novel environments, total genome size of five *Panicum* species, *P. capillare, P. decompositum, P. effusum, P. gilvum* and *P. hillmanii,* was determined using flow cytometric analysis of cells collected from fresh leaf tissue. Determination of genome size was based on coefficient of variation (CV) values below 10% (Fig. 4). The calculated genome size (1C value) of *P. capillare, P. decompositum, P. effusum, P. gilvum* and *P. hillmanii* was 1.24 pg, 1.49 pg and 1.52 pg, 0.21 pg and 0.24 pg, respectively, (Table 3). No significant differences in genome size were observed for samples of the same species collected from geographically distant locations.

**Table 3:** Flow cytometric analysis of genome size of *Panicum capillare, P. decompositum, P. effusum, P. gilvum* and *P. hillmanii* as estimated by comparison to *Raphanus sativus*, 1C = 0.55 pg or *Solanum lycopersicum,* 1C = 1.06 pg.

Taxonomic identification	Native or non-native	Genome size: 1C (pg)	Peak CV (%)
*P. capillare*	Non-native	1.24	4.40
*P. decompositum*	Native	1.49	3.61
*P. effusum*	Native	1.52	3.60
*P. gilvum*	Non-native	0.21	7.91
*P. hillmanii*	Non-native	0.24	6.83

## Discussion

Selection of the "barcode" for sequencing is critical when establishing a successful DNA barcoding approach or platform effective in differentiating individual *Panicum* species. An ideal barcode is typically a short DNA sequence that can be routinely amplified using a standard PCR method. The amplified product should be easily sequenced with universal primers, which are anchored in highly conserved DNA, and the sequence result should also be aligned without extensive manual editing^22^. Additionally, but most importantly, the barcode should strongly differentiate the *Panicum* species, and ideally, there should be no overlap between intraspecific and interspecific divergence^18,43^. Furthermore, the efficacy of any DNA barcoding methodology depends on the extent of differences between intraspecific and interspecific divergence in a selected locus or combined loci^43^. In our study, the *ITS* locus showed the highest minimum interspecific distance (0.71%), a distance that was significantly greater than the highest maximum intraspecific distance (*P. effusum*, 0.34%). This confirmed that *ITS* may be suitable as a standalone locus for the differentiation of selected *Panicum* species in Australia.

In contrast, we found significant overlap between intraspecific and interspecific distances for both *matK* and the *trnL* intron*-trnF* regions. Therefore, the individual application of either loci alone may be problematic for species differentiation in *Panicum* due to lack of intraspecific distance observed. However, the use of these loci in combination with *ITS* presents advantages when attempting to detect hybridization although there is currently no field or herbarium evidence of *Panicum* species hybridisation in southern New South Wales. Sequence combinations from nuclear and chloroplast genomes could provide additional information for enhanced species identification. For example, *trnL* intron*-trnF* shows the greatest prevalence among all noncoding chloroplast DNA sequences in GenBank to date^41^; and may assist in identification at the genus or species level in ambiguous specimens.

We compared the genotypic identification of indigenous and invasive *Panicum* species in Australia, and found that the native species *P. buncei, P. decompositum, P. effusum, P. laevinode* and *P. queenslandicum* were separated clearly from the non-native species *P. capillare, P. gilvum, P. hillmanii,* and *P. miliaceum*. These findings suggest that native Australian *Panicum* species have maintained a unique genetic fingerprint despite potential for hybridisation with non-native counterparts. Diversity in location-dependent accessions of *P. miliaceum* has recently been described, suggesting that genetic variation could be inherent at the population level^44^. This has potentially important implications for chemical or bioactive properties associated with this species. Interestingly, we noted that one non-native species, *P. coloratum*, was genetically more closely aligned with the native *P. buncei* than with non-native counterparts^45^*.* Further evolutionary analysis of these species, particularly with respect to correlating the molecular results with voucher specimens located in Australian herbaria, and those more globally, may be required to ensure correct identification.

The genome size of *P. gilvum*, *P. hillmanii, P. decompositum* and *P. effusum* has not previously been reported even though these species are frequently encountered across southern Australia. Genome sizes of *P. capillare*, *P. decompositum* and *P. effusum* were shown to be similar to ploidy size of other previously described *Panicum* species^42^. The genome sizes of *P. hillmanii* and *P. gilvum* were surprisingly smaller than predicted, and therefore we suggest a role for genome size in *Panicum* species identification and possibly prediction of invasive potential. Certain naturalised plants exhibit smaller genome size in contrast to their non-invasive or indigenous counterparts^42^, with the hypothesis that small genome size may confer biological advantage for adaptation in novel habitats, possibly due to enhanced tolerance of extreme environments or via altered regulatory gene divergence^35,46^. Given the challenging environmental conditions frequently encountered across inland Australia, and the successful establishment of these particular invasive grasses across southern Australia, the smaller genome size of the majority of non-native *Panicum* species investigated could be considered as supporting evidence for this hypothesis^47,48^.

Our results have shown that the use of the nuclear *ITS* region (and to a lesser extent the two cpDNA regions, namely *matK and trnL* intron*-trnF*) allowed clear identification and differentiation for eight of ten Panicum species evaluated, with only *P. buncei* and *P. coloratum* unable to be segregated using this method. We suggest that additional loci are likely required for further resolution at the species level, assuming the original taxonomic identification was correct. With the exception of *P. buncei*, discrimination between native and non-native species was achieved. Further studies to evaluate additional *Panicum* species from diverse habitats across Australia could confirm the utility of this approach. In addition to the techniques presented, other molecular tools, including whole or partial genome sequencing^49^, high resolution melt curve analysis^50^, short tandem repeats (STR)^51^, or some combination of the above, may prove useful for rapid and refined species differentiation through estimation of other genetic parameters.

In conclusion, this study reports the use of a DNA barcoding method for distinguishing field samples of *Panicum* species regardless of phenological growth stage, in isolation or in combination with traditional morphological identification. Rapid identification of *Panicum* grasses, including those commonly implicated in crop and pasture incursions^4^ or in hepatotoxicity outbreaks in livestock^7^, could assist producers, industry advisors, agronomists and weed scientists to identify invasive grasses accurately and quickly for control or eradication. This knowledge may also provide further insight into changing patterns of species distribution, and facilitate the development of efficacious weed management practices to limit invasive incursions or toxic outbreaks in pastures and croplands in Australia and internationally.

## Materials and methods

### Sampling

*Panicum* samples were collected within a 200 km radius of Wagga Wagga, New South Wales, Australia, in February–March 2017 and February–March 2018 when plants reached physiological maturity. Collection sites included roadsides, fallow croplands and pastures, and nature reserves, with a minimum distance between collection sites of 25 km. Permission for collecting non-threatened plant specimens was not required according to Biodiversity Conservation Act 2016 No 63, and verbal permissions have been given from the landowner if they were collected from private properties. Entire plants including inflorescences that exhibited visible morphological features of *Panicum* species were collected and stored at -20 °C. Whole *Panicum* plants were also collected at the reproductive phase and pressed for morphological identification and proper storage by a grass specialist and a co-author of this paper, David E. Albrecht, at the Australian National Herbarium (CANB). A small leaf section was collected from each plant and stored at -80 °C with silica gel to maintain tissue integrity before DNA extraction. In addition, fresh leaf tissue samples were also collected and stored at 4 °C for determining genome size using flow cytometry.

To supplement field-collected plant material, an additional 23 dried leaf samples representing nine previously identified *Panicum* species (Table 1), were sampled from voucher specimens held within the CANB collection (Acton, ACT, Australia). Dried leaf segments from archived plants of each of targeted species were provided by David E. Albrecht.

### DNA extraction and barcoding

Genomic DNA extraction was performed as described previously^52^. One nuclear DNA locus (*ITS*) and two chloroplast DNA loci (*matK* and *trnL* intron-*trnF*), were amplified by using MyTaq Red Mix (Bioline, Eveleigh, New South Wales, Australia). The following primer sets were used: ITS4 (TCCTCCGCTTATTGATATGC) and ITS5a (CCTTATCATTTAGAGGAAGGAG) for *ITS*^53^, 390F (CGATCTATTCATTCAATATTTC) and 1326R(TCTAGCACACGAAAGTCGAAGT) for matK^54^, ucp-c (CGAAATCGGTAGACGCTACG) and ucp-f (ATTTGAACTGGTGACACGAG ) for *trnL* intron*-trnF*^55^*.* Amplification conditions were 95 °C for 3 min, followed by 40 cycles of 95 °C for 30 s, 50 °C for 30 s, and 72 °C for 1 min, and a final extension at 72 °C for 5 min. PCR products were run on a 1.5% TAE agarose gel and stained using SYBRsafe (Invitrogen, Mulgrave, Victoria, Australia)^56^.

### Sanger sequencing and DNA barcode GAP analysis

PCR products were bidirectional Sanger sequenced using the same primers by the Australian Genome Research Facility, Brisbane. Sequences were read in Geneious version 11.0.5^57^. Forty-three sequences from each locus were aligned with a cost matrix of 65% similarity (Geneious version 11.0.5). Sequence alignments were analysed using MEGA7.0.26^58^ to calculate intraspecific and interspecific genetic distances with the Kimura 2-parameter (K2P) model. Sequences of three loci for each *Panicum* specimen were further concatenated for DNA barcode GAP analysis. Concatenated sequences of the same regions from *Setaria italica*, a member the tribe Paniceae, was used as an outgroup to root the tree. Phylogenetic relationships between species were inferred by MrBayes 3.2.6^59^ using default settings (four gamma categories, Markov chain Monte Carlo (MCMC) setting include chain length 1 million, subsampling every 1000th generation, burn-in length was first 250,000 iterations) with GTR substitution model for the nuclear DNA locus (*ITS*) and GTR + R substitution model for two concatenated chloroplast DNA loci (*matK* and *trnL* intron-*trnF*) as suggested by JModelTest 2.1.10^60^.

### Flow cytometry

Fresh leaf tissue was stored at 4 °C in moist paper towelling with cytometric analysis performed within 48 h using a Gallios Flow Cytometer (Beckman Coulter, USA). Depending on the species analysed, *Raphanus sativus* L. (red globe radish, 1C = 0.55 pg), or *Solanum lycopersicum* L. (tomato, 1C = 1.06 pg) were used as internal reference species for assessment of genome size. *R. sativus* was also used to calibrate *S. lycopersicum* within each run to confirm the reliability of each run. A composite leaf tissue sample of each targeted *Panicum* species and the reference plant, similar in size, were chopped using a clean razor blade in a premixed buffer solution, consisting of 1 ml WPB nuclear isolation buffer (0.2 M Tris. HCl, 4 mM MgCl_2_.6H2O, 2 mM EDTA Na_2_.2H_2_O, 86 mM NaCl, 10 mM sodium metabisulfite, 1% PVP-10, 1% (v/v) Triton X-100, pH 7.5)^61^, 50 μg propidium iodide (PI) (Sigma-Aldrich, Castle Hill, New South Wales, Australia), and 10 μl RNase A solution. At least 10,000 nuclei were analysed each run. Each specimen was analysed in triplicate with three technical replicates within 7 days of leaf collection to ensure reproducibility^62^.
